# Assessment of Thyroid Function and Oxidative Stress State in Foundry Workers Exposed to Lead

**DOI:** 10.5696/2156-9614-10.27.200903

**Published:** 2020-08-19

**Authors:** Yosri A. Fahim, Nevin E. Sharaf, Ibrahim W. Hasani, Eman A. Ragab, Heba K. Abdelhakim

**Affiliations:** 1 Egyptian Mineral Resources Authority (EMRA), Cairo, Egypt.; 2 Department of Environmental and Occupational Medicine, National Research Centre, Giza, Egypt.; 3 Department of Biochemistry, Faculty of Science, Idlip University and AL-Shamal Private University (SPU), Idlip, Syria.; 4 Department of Organic Chemistry, Faculty of Science, Cairo University, Giza, Egypt; 5 Department of Biochemistry, Faculty of Science, Cairo University, Giza, Egypt.

**Keywords:** foundry workers, thyroid hormones, oxidative stress, antioxidant, lead

## Abstract

**Background.:**

Exposure to lead (Pb) has been associated with endocrine, hematological, gastrointestinal, renal and neurological problems in humans. However, effects on the thyroid gland are controversial.

**Objectives.:**

The aim of the present study was to assess thyroid function in foundry workers occupationally exposed to Pb and the mechanism of oxidative-antioxidant imbalance.

**Methods.:**

Thyroid function parameters and markers of oxidative stress were examined in 59 adult males who had been occupationally exposed to Pb. The results were then compared to those of 28 male subjects who had no history of Pb exposure or thyroid abnormalities and served as a control group.

**Results.:**

Mean blood lead levels (16.5±1.74 μg/dl) were significantly higher among the exposed workers compared to those of the control group (12.8±1.16 μg/dl, (p <0.001)). The exposed group had significantly increased free triiodothyronine (FT3), free thyroxine (FT4) and significantly decreased thyroid stimulating hormone (TSH) (1.77±0.44 μIU/ml), whereas the control group had a TSH level of 2.61±0.94 μIU/ml (p< 0.0001). A state of oxidative stress was indicated by the significant increase in mean levels of malondialdehyde (MDA) and significant decrease in glutathione (GSH) (p < 0.0001). There was a significant positive correlation (r=0.358, p <0.05) between blood lead levels (BLL) and duration of employment, while BLL showed a significant negative correlation with TSH (r =−0.486, p <0.001), and GSH (r =−0.336, p <0.05). Of the occupationally exposed workers, 32.76% had elevated thyroid hormones. The results showed a significant positive relationship between GSH and TSH (β coefficient=0.274, p < 0.05), MDA with FT3 (β coefficient=0.355, p < 0.05) and FT4 (β coefficient = 0.491, p < 0.0001) among exposed workers.

**Conclusions.:**

Workers exposed to Pb dust proved to be at risk for hyperthyroidism, which was found to have a significant role in oxidative–antioxidant imbalance present among workers with increasing duration of exposure.

**Participant Consent.:**

Obtained

**Ethics Approval.:**

This study was approved by the Ethical Committee of the National Research Centre in Egypt (NRC) under the registration number 15225.

**Competing Interests.:**

The authors declare no competing financial interests.

## Introduction

Foundry processes involve pouring molten metal into a mold made to the external shape of the article to be cast. The mold may contain a refractory core which determines the dimensions of any internal cavity or hollow. Molten metal is then introduced into the mold. After cooling occurs, the mold is subjected to a ‘shake out' procedure which releases the casting and removes the core. The casting is then cleaned, and any extraneous metal is removed. Many changes have occurred in foundry technology and materials, but the basic processes and associated hazards have remained much the same in many foundries.^1^

During these processes, foundry workers may be exposed to hazards such as particulate matter and metals, silica, polycyclic aromatic hydrocarbons (PAH), high temperatures and machinery.^2^ Workers are mostly exposed to Pb through ingestion or inhalation. Although industrial foundries vary in terms of the type of metal being poured, the sand casting process, the type of furnace (induction, electric arc, and cupola) and finishing process (grinding, blast cleaning, and coating), the basic process and hazards including particles and metals remain the most significant occupational hazards in the foundry industry.^3^–^5^

In these environments, particulate matter is typically formed by metallic vapor condensation followed by oxidation reactions. Lead (Pb), mercury (Hg), cadmium (Cd) and zinc (Zn) are the main pollutants. Thus, foundry workers are exposed to different types of metals. Exposure to these metals results in pro-oxidant/antioxidant imbalance and can act as an intermediate in the formation of an oxidative stress state; as levels of the markers of lipid peroxidation malondialdehyde (MDA) increase and the activity of glutathione (GSH) enzyme decrease, the degree of oxidative stress could be affected.^6^–^9^

Occupational and chemical exposure might interfere with the hypothalamic pituitary thyroid axis at different levels and through different mechanisms of action.^10^ Studies of human populations have focused primarily on chemicals that are structurally similar to thyroid hormones such as polychlorinated biphenyls (PCBs), with little attention on heavy metals.^11^ Lead is known to have adverse neurological, hematological, renal, and gastrointestinal effects; however, associations with thyroid hormones have been inconsistent, and few occupational studies have examined associations with thyroxine (T_4_), free thyroxine (FT_4_), triiodothyronine (T_3_), free triiodothyronine (FT_3_), or thyroid stimulating hormone (TSH).^12^–^16^ The present study was designed to assess thyroid function and presence of an oxidative stress state among foundry workers occupationally exposed to Pb dust and fumes.

## Methods

The present work was a cross sectional comparative study conducted in a non-ferrous foundry plant in Helwan, Cairo, Egypt. The foundry plant manufactures aluminum, Pb, Zn, copper and precious metal products.

**Abbreviations**
BLL: Blood lead level
FT_3_: Free triiodothyronine
FT_4_: Free thyroxine
GSH: Glutathione
MDA: Malondialdehyde
ROS: Reactive oxygen species
T_3_: Triiodothyronine
T_4_: Thyroxine
TSH: Thyroid stimulating hormone

The present study was conducted from April 2016 to May 2017.

### Study population

The study population was comprised of the work force in the Pb casting departments. After applying the exclusion criteria, which included workers who have undergone thyroid surgery or receiving any form of thyroid treatment, 61 workers were eligible for inclusion and only two workers did not agree to participate in the study. Another group of referents (n=28) were randomly selected from men employed at administrative jobs who lived in residential areas away from the factory and were never occupationally exposed to metals. Both groups were matched for age, social economic status and smoking habits. All subjects were interviewed using a questionnaire involving occupational history, and clinical examination, including thyroid gland inspection and palpation, was performed by a specialized physician. The questionnaire can be found in Supplemental Material.

This study was approved by the Ethics Committee of the National Research Centre in Egypt (NRC) under the registration number 15225.

### Blood collection

Blood samples were collected from all subjects using a dry plastic disposable syringe and divided into two parts: the first part (3 ml) was collected into K-EDTA tubes for Pb and GSH evaluation, the second part (3 ml) was collected into serum vacationer tubes for thyroid hormones and MDA measurements.

## Analytical methods

Lead level evaluation was performed using the simultaneous inductively coupled plasma emission spectrometer (Agilent 720 ICP-OES) and the method described by Momen *et al*.^17^

Quantitative measurements of serum FT_3_, FT_4_ and TSH were carried out using an enzyme immunoassay kit purchased from International Immuno-Diagnostics Co., USA (Gamma Trade Company), and the methods described by Melmed *et al*., Tarnoky, and Synder *et al*.^18^–^20^

Quantitative determination of serum MDA was carried out calorimetrically using a kit purchased from Biodiagnostic Co., Egypt, according to the method described by Ohkawa *et al*.^21^ Blood glutathione levels were estimated using a kit from Biodiagnostic Co., Egypt, according to the method described by Beutler *et al*.^22^

Statistical evaluation of all results was conducted using the Statistical Package for the Social Sciences software (SPSS) version 16. The mean values, SDs and ranges were estimated for quantitative variables. The correlations between individual variables were calculated using Pearson correlation coefficient. *P-*values <0.05 were considered statistically significant. Multiple linear regression analysis was used to estimate the influence of independent variables such as GSH and MDA on the markers studied (dependent variables).

## Results

The exposed group had an age range of 28–59 years with a mean value of 44.9±10.36 years, which did not differ significantly compared to the control group. Smoking habits showed no significant difference between the two study groups. Mean blood lead levels (BLL) were significantly higher among the exposed workers (16.5±1.74 μg/dl) compared to the control group (12.8±1.16 μg/dl) (*p* <0.0001). Their mean duration of employment was 22.35±10.7 years. The exposed group showed a significant increase in thyroid hormones (FT_3_, FT_4_) compared to the control group (*p* <0.0001). In addition, TSH mean value (1.77±0.44 μIU/ml) was significantly decreased compared the control group (2.61±0.94 μIU/ml). The exposed group also showed a state of oxidative stress represented by a significant increase in mean levels of MDA and a significant decrease in mean levels of GSH (*p* <0.0001) *(Table 1).*

**Table 1:** Characteristic of Study Subjects

**Parameters**	**Exposed (n= 59)**	**Control (n=28)**	***p*-value**
Age (years)	44.9±10.36	41.36±7.33	0.108
Duration of employment (years)	22.35±10.7	-	-
Non-smoker [n (%)]	30 (50.9)	19 (67.86)	0.135
Smoker [n (%)]	29 (49.1)	9 (32.14)	
BLL (μg/dl)	16.5±1.74	12.8±1.16	0.0001
MDA (nmol/1)	16.87±3.97	9.95±1.73	0.0001
GSH (mg/dl)	18.76±5.05	29.02±4.20	0.0001
MDA/GSH	0.96±0.35	0.35±0.08	0.0001
TSH (μIU/ml)	1.77±0.44	2.61±0.94	0.0001
FT_3_ (pg/ml)	4.23±1.82	2.19±0.77	0.0001
FT_4_(ng/dl)	1.97±0.55	1.42±0.48	0.0001

In the correlation of BLL with duration of employment, there was a statistically significant positive correlation (r=0.358, *p* <0.05) and a negative correlation with TSH (r =−0.486, *p* <0.001), and GSH (r =−0.336, *p* <0.05) *(Table 2).*

**Table 2:** Correlation Coefficient of Duration of Employment, Thyroid Hormone Level and Oxidative Stress Biomarkers with BLL (n= 47) Among Exposed Workers

		**BLL (μg/dl)**
Duration of employment	r	0.358
	p	0.013
MDA (nmol/1)	r	0.034
	p	0.823
GSH (mg/dl)	r	−0.336
	p	0.021
TSH (μIU/ml)	r	−0.486
	p	0.001
FT_3_ (pg/ml)	r	0.102
	p	0.496
FT_4_(ng/dl)	r	0.256
	p	0.496

Using the independent t-test to compare the mean value of oxidative stress biomarkers between exposed workers with normal FT_3_, FT_4_, and elevated FT_3_, FT_4_, the present study found that the MDA mean value was very significantly elevated in exposed workers with elevated thyroid hormones (*p* <0.001). Exposed workers with elevated thyroid hormones represented 32.76% of exposed workers *(Table 3).*

**Table 3:** Comparison of Oxidative Stress Markers Between Workers with Normal and Elevated FT_3_ and FT_4_ Among the Exposed Group

	**Normal FT_3_ and FT_4_ (40) mean ±SD**	**Abnormal elevated FT_3_ and FT_4_ (19) mean ±SD**	**t-test**	**p-value**
MDA (nmol/1)	15.22±2.33	20.36±4.48	−5.80	0.0001
GSH (mg/dl)	19.28±5.43	18.51±4.91	−0.54	0.590
MDA/GSH	0.88±0.26	1.15±0.44	−2.90	0.005

Multiple linear regression analysis was applied with MDA or GSH as dependent variables, using TSH, FT_3_ and FT_4_ as independent variables. The results showed a significant positive relationship between GSH and TSH, and between MDA and FT_3_ and FT_4_ among exposed workers *(Table 4).*

**Table 4:** Association Between Various Oxidative Stress Markers and Thyroid Profiles by Multiple Linear Regression Analysis (n= 46)

**Dependent variable**	**Independent variable**	**β coefficient**	**t-test**	**p-value**
GSH (mg/dl)	TSH (μIU/ml)	0.274	2.15	0.036
MDA (nmol/1)	FT_3_ (pg/ml)	0.355	2.80	0.007
	FT_4_ (ng/dl)	0.491	3.88	0.0001

## Discussion

Occupational exposure to heavy metals can cause many harmful health effects, depending on the intensity and duration of exposure. Many studies have suggested that occupational and environmental exposure to heavy metals such as Hg, Cd, chromium, arsenic, nickel and Pb cause oxidative damage and are capable of disrupting the activity of several proteins in the reproductive and endocrine system.^7^,^9^,^23^–^27^

Oxidative stress is a well-documented mechanism of metal toxicity and carcinogenicity. It is the result of imbalance between radical oxygen species production and the antioxidant defense system. Redox-inactive toxic metals such as Pb deplete cells of antioxidant reserves, especially GSH, which plays a pivotal role in its overall toxic manifestations.^7^–^9^ Heavy metals are found in the air of nonferrous alloy foundries, because they are released as fumes during the alloy manufacturing process.^4^ Whole blood has been the primary biological fluid used for the assessment of Pb exposure, both for screening, diagnosis and for long-term bio-monitoring.^28^

The characteristics of the study population are summarized in Table 1. For the exposed group, the obtained BLL value was 16.5±1.74 μg/dL, which is below the Occupational Safety and Health Administration (OSHA) Pb standard (50 μg/dL), however the OSHA Pb standard is from the 1970's when BLLs were much higher, and is considered outdated.^29^ The long duration of exposure may have a large influence on worker BLLs and cumulative levels over extended periods of time can pose health risks, which goes with our findings of a positive correlation between the duration of exposure and elevated B-Pb levels. The relationship between BLL and duration of employment was highly significant (r=0.358, *p* <0.05), which indicates that work activity had a direct relationship with the risk of occupational exposure to Pb and indicates an increased body burden of Pb among the exposed workers due to their occupational setting.

Some metals have the ability to produce reactive oxygen species (ROS) in biological systems, which may lead to a state of oxidative stress. It is a state where increased formation of ROS overwhelms the body's antioxidant production and subsequently induces lipid peroxidation, DNA damage, protein modification and other effects. The underlying mechanism of these toxic metals involves the production of MDA and depletion of GSH. Lead may induce oxidative stress that may deteriorate biological macromolecules either by increased ROS or depletion of a cell's major antioxidants.^7^,^30^

The present study found that the exposed group suffered from a state of oxidative stress, indicated by significantly higher serum MDA and a significant lower GSH in the exposed group compared to the controls. The higher MDA level is consistent with the results of Liu *et al*. and Sciskalska *et al*.^3^,^31^ Lowered GSH has been demonstrated in many experimental studies in which rats were exposed to Pb.^32^,^33^ In the present study, foundry workers exposed to Pb showed significantly decreased TSH mean levels, and a significant increase in FT_3_ and FT_4_ mean levels compared to the control group. Lead is known to have adverse neurological, hematological, renal and gastrointestinal effects. However, associations with thyroid hormones have been inconsistent.^14^

Yilmaz *et al*. found that FT_3_ levels were significantly higher in subjects with Pb exposure compared to the control group (*p* <0.01), TSH levels were lower (*p* < 0.001), but no significant difference was found for FT_4_ between the two groups.^34^ Other studies have shown statistically significant elevation of FT_4_ and non-significant reduction of TSH in Pb-exposed workers compared with controls.^35^ One study found a dramatic decrease of TSH in a Pb-exposed group and a decrease in T_3_ and T_4_.^36^ Another occupational study showed that workers exposed to Pb had significantly higher TSH than controls and non-significant higher levels for thyroid hormones FT_3_ and FT_4._^15^

Lead is a redox-inactive metal, it depletes cells' major antioxidant reserves, especially GSH, which plays a pivotal role in its overall toxic manifestations.^7^–^9^ Cells have developed various antioxidant systems against free radical attacks. Glutathione plays a major role in protecting cells against oxidative stress. The glutathione functional group (sulfhydryl group) plays an important role in metal binding. Several studies have demonstrated decreased GSH levels in rats exposed to Pb.^32^,^37^

The disulphide bond is found in the active site of glutathione reductase, the disulphide bond interferes with Pb and inhibits enzymes. The inhibition prevents glutathione disulfide from being reduced to GSH, thus Pb deplete cells' major antioxidants, enhanced generation of ROS and results in an oxidative stress state.^7^ This is in agreement with the results of the present study which found a negative correlation between BLL and the marker of oxidative stress, GSH (r=−0.336, *p* <0.05). Our results are in agreement with those of many studies which suggests that Pb is a redox inactive metal that induces oxidative stress in cells and can be partially responsible for its toxicity.^32^,^38^,^39^ All cited studies have shown a decrease in GSH levels during Pb toxicity, similar to our study. Previous studies have investigated oxidative damage as a possible mechanism involved in Pb toxicity and found that GSH was significantly increased compared to control groups.^40^

The present study reported a negative association between BLL and TSH (r= −486, *p* <0.001) and no association with FT_3_ and FT_4_, although they were significantly higher in the exposed group compared to the control. Previous studies have suggested the same association among males and females, while others found negative associations with FT_3_ and FT_4_, however, associations were not evident in other similar studies.^14^,^41^–^46^

Paint workers exposed to Pb and solvents were at risk for hyperthyroidism, as T_3_ and T_4_ were significantly higher in workers compared to controls,^10^ similar to the present study. Additionally, the authors found that T_3_ significantly correlated with MDA in paint workers. In addition, MDA was significantly higher in workers with elevated T_4_, again, similar to the present study, which found that MDA was highly significant in exposed workers with elevated FT_3_ and FT_4_ (*p* <0.001).^10^ In addition, a multiple linear regression analysis was applied with MDA or GSH as dependent variables and using TSH, FT_3_ and FT_4_ as independent variables. A positive association between GSH and TSH (*β* coefficient =0.274, *p* <0.05), and MDA with FT3 (*β* coefficient =0.355, *p* <0.05) and FT_4_ (*β* coefficient =0.491, *p* <0.0001) was observed among exposed workers.

## Conclusions

The results of the present study suggest that occupational exposure to Pb dust and fumes has a stimulatory effect on thyroid function as manifested by a significant increase in thyroid hormone levels, even if this increase was not associated with clinical manifestations of hyperthyroidism. Foundry workers exhibited an increase in MDA levels and decrease in GSH levels, which represents evidence for oxidative stress imbalance, in which increased thyroid hormones play a significant role in ROS production through stimulation of metabolism and the increase of MDA levels. In addition, exposure to Pb, a redox inactive metal, depletes cells' major antioxidants reserves of GSH. Mounting evidence indicates that multiple mechanisms may be responsible for the oxidative stress imbalance caused by exposure to toxic metals such as Pb, including thyroid stimulation.
