# Supplementary material for: Assessment of Thyroid Function and Oxidative Stress State in Foundry Workers Exposed to Lead
Source: J Health Pollut. 2020 Aug 19;10(27):200903. doi: 10.5696/2156-9614-10.27.200903 (PMC7453815; doi:10.5696/2156-9614-10.27.200903)
Supplement: Supplementary file 1 [file Fahim_Supplemental.docx]

**Supplemental Material**

**Questionnaire Form**

| Full name: | Serial no.: Age: |
| --- | --- |
| Marital status: | Currently living: |
| Type of job: | Sector: |
| Duration of employment: | Ex-job: |
| Exposure hours: | Smoking habit:  Yes: No: Ex-smoker: |
| Usage of personal protective equipment:  Mask: Gloves: Other: | |
| Medicinal drugs:  No: Yes: Kinds of drugs: | |
| Alcohol abuse:  No: Yes: Duration: | |
| Genetic disorders or history of severe medical diseases:  No: Yes: What kind:  Cardiovascular:  Renal:  Hepatic disorders:  Diabetes mellitus:  Cancer: | |
| Complains: | |
| Physician report: | |
